# Cordycepin (3′dA) Induces Cell Death of AC133^+^ Leukemia Cells via Re-Expression of *WIF1* and Down-Modulation of *MYC*

**DOI:** 10.3390/cancers15153931

**Published:** 2023-08-02

**Authors:** Nazanin Abazari, Marta Rachele Stefanucci, Luca Emanuele Bossi, Alessandra Trojani, Roberto Cairoli, Alessandro Beghini

**Affiliations:** 1 Department of Health Sciences, University of Milan, 20142 Milan, Italy; nazanin.abazari@unimi.it (N.A.); martarachele.stefanucci@ospedaleniguarda.it (M.R.S.); 2 Department of Hematology and Oncology, ASST Grande Ospedale Metropolitano Niguarda, 20162 Milan, Italy; lucaemanuele.bossi@ospedaleniguarda.it (L.E.B.); alessandra.trojani@ospedaleniguarda.it (A.T.); roberto.cairoli@ospedaleniguarda.it (R.C.)

**Keywords:** AML, cordycepin, WIF1, MYC, AC133^+^

## Abstract

**Simple Summary:**

Cordycepin, an analog of adenosine, has shown anti-leukemic properties towards leukemic stem cells (LSCs) by perturbing the leukemia−stroma association. However, the full molecular picture has not been fully elucidated. In addition, Wnt/β-catenin signaling is required for LSC development and maintenance in acute myeloid leukemia (AML). Here we show that cordycepin downregulates the Wnt target genes *MYC* and *PROM1* (CD133), critical factors in stem cell maintenance, via re-expression of WIF1 and DKK1. The results provided offer new insights into the molecular circuits involved in the cordycepin-mediated inhibition of Wnt signaling. This mechanism of action of cordycepin has potential applications in treating AML.

**Abstract:**

Wnt/β-catenin signaling is critically required for the development and maintenance of leukemia stem cells (LSCs) in acute myeloid leukemia (AML) by constitutive activation of myeloid regeneration-related pathways. Cell-intrinsic activation of canonical Wnt signaling propagates in the nucleus by β-catenin translocation, where it induces expression of target oncogenes such as *JUN*, *MYC* and *CCND1*. As the Wnt/β−catenin pathway is now well established to be a key oncogenic signaling pathway promoting leukemic myelopoiesis, targeting it would be an effective strategy to impair LSC functionality. Although the effects of the adenosine analogue cordycepin in repressing β-catenins and destabilizing the LSC niche have been highlighted, the cellular and molecular effects on AML–LSC have not been fully clarified. In the present study, we evaluated the potency and efficacy of cordycepin, a selective repressor of Wnt/β-catenin signaling with anti-leukemia properties, on the AC133^+^ LSC fraction. Cordycepin effectively reduces cell viability of the AC133^+^ LSCs in the MUTZ−2 cell model and patient-derived cells through the induction of apoptosis. By Wnt-targeted RNA sequencing panel, we highlighted the re-expression of WIF1 and DKK1 among others, and the consequent downregulation of MYC and PROM1 (CD133) following MUTZ−2 cell exposure to increasing doses of cordycepin. Our results provide new insights into the molecular circuits involved in pharmacological inhibition mediated by cordycepin reinforcing the potential of targeting the Wnt/β-catenin and co-regulatory complexes in AML.

## 1. Introduction

Our understanding of leukemogenesis has evolved considerably in the last decade, revealing that transformed leukemic stem cells (LSCs) are functionally related to the CD133^+^CD34^+^CD38^-^lymphoid-primed multipotential progenitors (LMPPs) [1,2,3]. LMPP-like LSCs continuously produce CD34^+^38^+^ granulocyte/macrophage progenitor (GMP)-like cells [1,4] as a direct consequence of constitutive activation in driving networks, such as the regeneration-associated Wnt/β-catenin signaling in bone marrow (BM) niche [2,5,6,7,8,9]. Cell-intrinsic Wnt/β-catenin signaling hyperactivation is thought to induce inappropriate expression of target genes controlled by the Wnt responsive DNA elements (WREs) such as *MYC*, *JUN*, *CD44* and *CCND1,* which predict enhanced clonogenic potential and unfavorable prognosis [10,11,12,13,14,15,16,17].

MYC is required for adult hematopoiesis [18,19,20] and plays a central role in G1/S transition as an upstream modulator of cell cycle regulatory molecules [21]. MYC haploinsufficiency led to ineffective hematopoiesis by inhibiting HSC self-renewal and quiescence and by promoting apoptosis [22]. Thus, MYC is critical for balanced hematopoiesis and is frequently overexpressed in acute myeloid leukemia, likely by oncogenic Wnt/β-catenin signaling through the specific WRE associated with the *MYC* promoter (*MYC* 5′ WRE), leading to proliferation and terminal differentiation impairment of HSCs [16,23,24,25,26].

Furthermore, activation of the Wnt signaling pathway is required for the survival and development of LSCs [5] and has been implicated in aberrant methylation of Wnt antagonists [27], such as Wnt inhibitory factor 1 (*WIF1*) and Dickkopf-1 (*DKK1*) in acute myeloid leukemia [28]. Targeting Wnt signaling in leukemia represents an attractive therapeutic strategy to suppress the leukemia-initiating properties of the CD44^+^CD133^+^ cells [29]. However, the lack of effective modulatory agents has limited this approach to date [30]. In recent years, the anti-leukemia properties of cordycepin (also known as 3-deoxyadenosine), a major active component of the fungi *Cordyceps militaris* and *C. sinensis*, have been suggested by the impairment of Wnt/β-catenin signaling at different levels [31,32,33]. Therefore, the purpose of the present study was to elucidate the molecular circuits involved in cordycepin-mediated suppression of Wnt signaling in AC133^+^ leukemia cells. Our findings suggest that cordycepin induces apoptosis through different levels of action; in particular, we highlight the downregulation of MYC and PROM1 (CD133) mediated by the re-expression of WIF1 and DKK1.

MYC represents a complex problem in the control of cell proliferation, as it participates in the regulation of all hallmarks of cancer [34]. The effective MYC down-modulation demonstrated by cordycepin suggests the importance of further investigating its potential use as an antileukemic adjuvant.

## 2. Materials and Methods

### 2.1. Cell Line and Primary Cell Cultures

AC133^+^-MUTZ-2 cells (DSMZ ACC 271) were grown in 60% alpha-MEM (Gibco) medium, complemented with 20% fetal bovine serum (FBS), 20% vol conditioned medium by 5637 cell line (DSM ACC 35), 1% penicillin/streptomycin, 1% of glutamine and 50 ng/mL SCF (STEM CELL Technologies). All cells were grown at 37 °C in a humidified incubator (Binder) with 5% CO_2_. Cell lines were routinely tested to exclude mycoplasma contamination. Primary cell cultures originated from mononuclear cells obtained from patient AML#526, and AC133^+^ cells were selected from patient AML#523. Patient samples were collected at the Department of Hematology, ASST Grande Ospedale Metropolitano Niguarda of Milan, with informed consent of the subjects. Primary cell cultures were kept in an incubator at 37 °C and with 5% CO_2_ for 4 days before the experiment. The medium was composed of 55% Alpha-MEM, 20% FBS, 25% vol conditioned medium by 5637 cell line (DSM ACC 35), 1% of penicillin/streptomycin, 1% of glutamine and 50 ng/mL of SCF.

### 2.2. Cell-Based Assays

MUTZ-2 cell line and primary cells were treated with different concentrations of cordycepin (Sigma, Sofia, Bulgaria). The viability of cells after concentration and time-dependent treatments was determined using both manual cell counting by 0.4% Trypan Blue staining (Sigma Aldrich) and the standard MTT [3-(4,5-dimethylthiazol-2-yl)-2,5-diphenyltetrazolium bromide] metabolic activity assay (Sigma Aldrich), performed in 96-well plates. Cell viability was measured by a methyl thiazole tetrazolium (MTT) assay for 6 and 24 h. At the end of each culture period, 10 µL (1:10) of a 5 mg/mL solution of MTT was added to each well, followed by incubation at 37 °C for 3 h. Metabolically active, viable cells converted MTT into a colored formazan, which was made soluble with a volume of 0.1 N HCl in anhydrous isopropanol equal to the volume of cell suspension (100 μL). Cell viability was evaluated by measuring the absorbance at 570 nm, using an absorbance reader (Multi-mode plate reader, PerkinElmer). The viability was expressed as the percentage of optical density of treated cells compared to optical density of cells treated with the specific vehicle. Each experimental condition was done in hexaplicate and repeated at least twice. Apoptosis of the MUTZ-2 cell line was assessed by labeling cells with an eBioscience™ Annexin V-FITC Apoptosis Detection Kit (Thermofisher). MUTZ−2 cells were seeded in 12-well plates and treated with different concentrations of cordycepin (20, 50 and 100 μM), for 48 and 72 h. The cells were then washed twice with PBS 1X and resuspended in a binding buffer containing (1:40) Annexin V-FITC. All conditions were incubated for 15 min at room temperature in a light-protected area, then cells were washed with binding buffer and resuspended in a binding buffer containing 10 μL propidium iodide (20 μg/mL), and then analyzed by flow cytometry (FACS Calibur; Becton Dickinson). The results were analyzed with FACS, setting the excitation wavelength at 488 nm for both the dyes and emission fixed at 530 nm for FITC and 655–730 nm for PI. Data were analyzed using FlowJo software (v10, Treestar, Inc., San Carlos, CA, USA).

### 2.3. Transcriptomic Analysis

Total RNA was isolated from MUTZ-2 using an isolation Kit (ThermoFisher Scientific) according to the manufacturer’s instructions. The quality and the yield of the extracted RNA was evaluated using a Qubit 4 fluorometer in combination with an RNA HS Assay Kit (ThermoFisher Scientific Inc., Waltham, MA, USA). RNA (200 ng) was reverse transcribed, using an ImProm-II reverse transcription system (Promega, Tokyo, Japan) and 0.5 µg/reaction of random primer (Promega), according to the manufacturer’s instructions. All samples within an experiment were reverse-transcribed at the same time, followed by PCR endpoint utilizing Platinum™ Hot Start PCR Master Mix 1× and 0.5 μM of forward and reverse WIF1 primers (Table S1) using 50 ng cDNA in a total volume of 20 μL. The amplifications of WIF1 were performed as follows: 94 °C for 2 min, (94 °C for 15 s, 56 °C for 15 s and 72 °C for 15 s) for 33 cycles and 4 °C 10 min. The PCR products were then analyzed by electrophoresis through a 2% agarose gel.

### 2.4. Real-Time qPCR Analysis

Real-time qPCR was carried out using PowerUp^TM^ SYBR^TM^ Green Master Mix, according to the manufacturer’s protocols. All samples (10 ng cDNA) were analyzed in triplicate. The expression of MYC and WIF1 was normalized to the most stable reference gene glyceraldehyde 3-phosphate dehydrogenase (GAPDH) by the StepOnePlus™ Real-Time PCR System, and data were analyzed using the 2^−ΔΔCt^ methods. PCR primers are detailed in Table S1. Oligonucleotides for qPCR were designed using Primer 3 (https://bioinfo.ut.ee/?page_id=163&lang=en, version 0.4).

### 2.5. Ion AmpliSeq NGS WNT-Panel for Targeted Sequencing

Next-generation sequencing (NGS) for RNA analysis was performed on MUTZ-2 treated in triplicates with different concentrations of cordycepin (50 µM, 100 µM and 200 µM) for 6 and 24 h, using a custom 179 gene Ion Ampliseq panel which was based on Ion Ampliseq RNA Wnt signaling panel (ThermoFisher Scientific) with additional amplicons (Panel WG_IAD195199.20200413). The additional genes analyzed were: MARK4 (NM_001199867), NDP (NM_000266), PROM1 (NM_001145847), MLLT11 (NM_006818), WNK2 (NM_006648), TBL1XR1 (NM_024665), ROCK2 (NM_004850), LRRFIP2 (NM_001134369) and USP34 (NM_014709). RNA concentration was evaluated using a QubitTM 4 fluorometer in combination with an RNA HS Assay Kit (ThermoFisher Scientific). Library preparation was performed on an Ion Chef™ instrument following the AmpliSeq™ Kit for the Chef DL8 protocol (ThermoFisher Scientific #A29025) for automated preparation of libraries. Enriched samples were sequenced on the Ion S5 System Instrument using a 530 chip. Sequencing results were preliminarily analyzed using Ion Torrent Suite 5.12.1. Coverage analysis was performed using plugin AmpliseqRNA 5.12.0.1 to map the sequencing reads on target regions. The reference library used was hg19 Ampliseq Transcriptome. Data analysis was performed with the Transcriptome Analysis Console 4.0 software (TAC). We examined the differential expression in all conditions, maintaining the subdivision by concentration and hours of treatment. We compared the expression at each concentration to the untreated control. Finally, we considered as significantly differentially expressed (SDE) genes those genes having a *p*-value lower than 0.05, further selecting those genes with a negative exponential value and an absolute log2FC value higher than or equal to 2. Fold-change value can be used to estimate how many times a gene is up- or downregulated.

### 2.6. Immunoblot

The MUTZ-2 cells were treated with different concentrations of cordycepin (50, 100 and 200 μM) for 48 and 72 h. Total protein was extracted using RIPA buffer containing 100 mM Tris (pH 7.6), 1% Triton X-100, 150 mM NaCl, 2 mM PMSF, 10 mM Na3VO4, 100 mM NaF, 10 mM Na4P2O7 and 4 mM EDTA.

Protein fractions were separated by 10% SDS gel-electrophoresis and were transferred to a polyvinylidene difluoride membrane by semi-dry blotting, following the protocols from the manufacturer (Bio-Rad, Hercules, CA, USA). After overnight incubation with 5% non-fat dry milk (NFDM) in TBS-T (10 mM Tris pH 7.5, 100 mM NaCl, 0,1% Tween-20), the membrane was incubated with primary antibodies against MYC (1:1000, BETHYL) and GAPDH (1:1000, BETHYL) at 4 °C overnight. Membranes were washed 3 times for 10 min prior to incubation with the secondary antibody anti-rabbit HRP (1:5000, ThermoFisher Scientific) for 30 min. Blots were washed 3 times with TBS-T and detected by enhanced chemiluminescence reagents (Clarity Western ECL, Bio-Rad).

The *MYC* gene encodes a polypeptide with a predicted molecular weight of 49 kDa but also demonstrates aberrant electrophoretic mobility in Western blotting to manifest an apparent molecular weight of around 62 kDa (p62c-myc).

Data normalization is required to accurately compare target protein expression across multiple samples in immunoblot analysis. Images were acquired on ChemiDoc MP Instrument (Bio-Rad) by Image Lab Touch Software (2.4). To normalize target protein expression, the band intensity of each sample is determined by densitometry analysis performed using Image Lab Software (Bio-Rad). Then, the intensity of the target protein is divided by the intensity of the loading control protein. This calculation adjusts the expression of the protein of interest to a common scale and reduces the impact of sample-to-sample variation. Relative target protein expression can then be compared across all lanes to assess changes in target protein expression across samples calculating the fold change by dividing the normalized expression from each lane by the normalized expression of the control sample in the first lane.

### 2.7. Statistical Analysis

Data are presented as mean ± SEM or ratios among treatments and controls, in independent experiments as indicated in the legends of the figures. Statistical analyses were performed using GraphPad Prism 9 (GraphPad Software, USA; Biomatters, Ltd., NZ; and GSL Biotech, USA). ANOVA tests and ANOVA and Tukey’s multiple comparisons test were used for comparisons. A *p*-value < 0.05 was considered statistically significant.

## 3. Results

### 3.1. Modulation of Cell Viability after Treatment with Cordycepin on MUTZ-2 and Primary Cells

**Figure 1:**
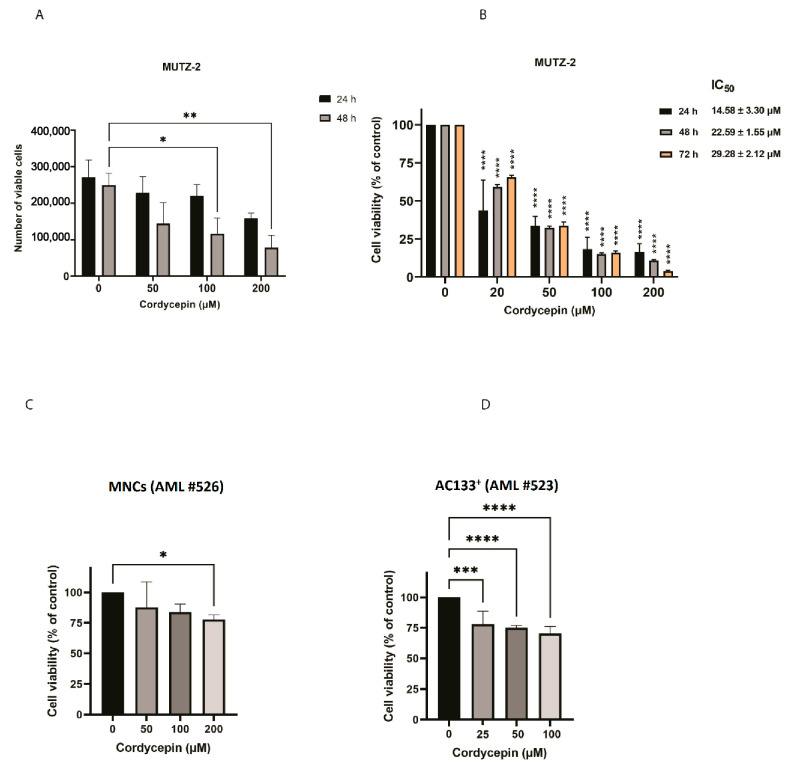
Effects of exposure to increasing concentration of cordycepin on MUTZ−2 and primary AML cell viability. (**A**) Cell viability was analyzed through Trypan blue cell count in MUTZ−2 cells after cordycepin treatment at increasing concentrations (50, 100, 200 µM) for 24 and 48 h. (**B**) Dose- and time-response cytotoxicity of the drug were analyzed by the methyl thiazole tetrazolium (MTT) assay in MUTZ−2 cells treated with increasing concentrations of cordycepin (20, 50, 100 and 200 μM) for 24, 48 and 72 h. Values are expressed as the percentage of viable cells for each condition relative to controls. (**C**) MTT assay on mononuclear cells (MNCs) from an AML patient (AML#526) with increasing concentrations of cordycepin (50, 100 and 200 μM) for 24 h. (**D**) MTT assay on primary selected cells (AC133^+^) from an AML patient (AML#523) with increasing concentrations of cordycepin (25, 50 and 100 μM) for 24 h. The relative IC_50_ for MUTZ−2 is shown as the mean ± SD (standard deviation) of at least three independent experiments; the *p*-values are indicated in the graphs: * *p* < 0.05; ** *p* < 0.01; *** *p* < 0.001; **** *p* < 0.0001; ANOVA and Tukey’s multiple comparisons test.

With the aim of evaluating the inhibitory effect of cordycepin on the Wnt signal in the AC133^+^ leukemic stem component, we initially evaluated the MUTZ-2 cell line as the only acute myeloid leukemia cell line enriched for the AC133^+^ marker [35]. Treatment of the MUTZ-2 cell line with different concentrations of cordycepin (50, 100, 200 µM) and times (24, 48, 72 h) revealed that cordycepin can significantly reduce the cell viability in a dose-dependent manner. We performed two different assays, including trypan blue for manual cell counting (Figure 1A) and the methyl thiazole tetrazolium (MTT) assay in MUTZ−2 cells (also including 20 µM of cordycepin) (Figure 1B). MTT results show relative IC_50_ values of 14.58 ± 3.30, 22.59 ± 1.55 and 29.28 ± 2.12 µM, with R-squared values of 0.8887, 0.9956 and 0.9956, respectively, for 24, 48 and 72 h. These results indicate a remarkable drug effect even at 24 h after treatment, showing a 56.1% reduction in cell viability at 20 μM of cordycepin (Figure 1B).

In addition, to confirm the results obtained for the MUTZ−2 cell line, cordycepin was also tested on primary cells from different AML patients. The reduction in cell viability was milder in mononuclear cells (MNCs) derived from patient AML#526 showing 77.68% viability at the highest dose (200 µM) after 24 h (Figure 1C). Cordycepin was more effective on AC133^+^ primary cells (AML#523) with *p* < 0.0001, compared to the MNCs. Analysis of AC133^+^ cells indicates that these cells were more sensitive to this drug, with a 29.65% reduction in cell viability at the 100 µM dose after 24 h (Figure 1D).

### 3.2. Cordycepin Induces High Apoptosis Rates in MUTZ-2 Cells

**Figure 2:**
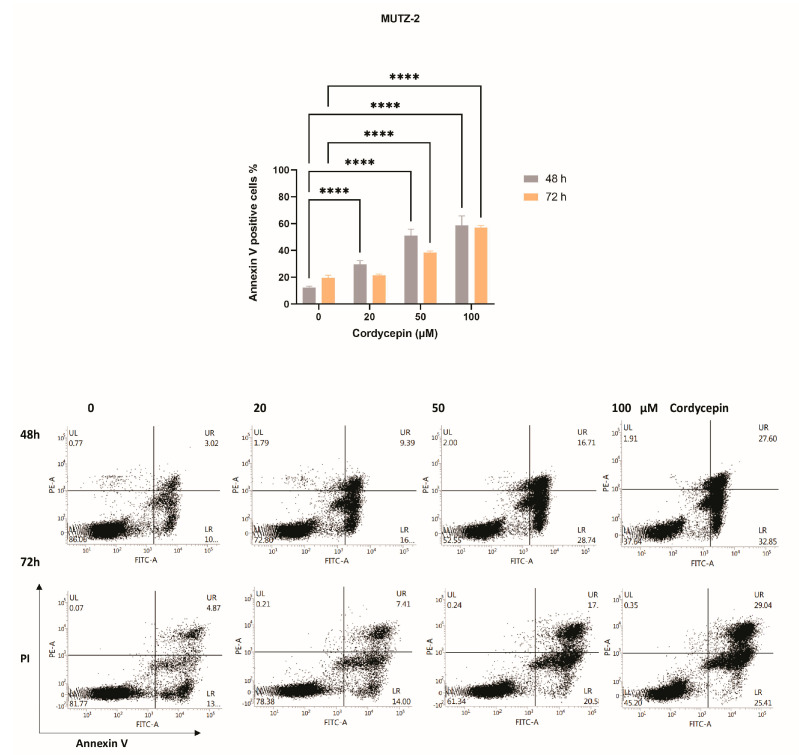
Effects of cordycepin on apoptosis in MUTZ−2. (**Upper panel**) Apoptosis was detected by flow cytometry and the Annexin V/propidium iodide (PI) staining method in MUTZ−2 cells treated with growing concentrations of cordycepin for 48 and 72 h. Bar graphs represent the mean ± SD of at least three independent experiments. The *p-*values and cell lines are indicated in the graphs: **** *p* < 0.0001; 2−way ANOVA and Tukey’s multiple comparisons test. (**Lower panel**) Representative dot plots are shown for each condition; the upper and lower right quadrants (UR plus LR) cumulatively contain the apoptotic population (Annexin V+ cells).

Evaluation of apoptosis using Annexin V and PI labeling revealed that cordycepin can induce significant levels of apoptosis (*p* < 0.0001) by increasing the drug dosage (20, 50, 100 µM) in a dose-dependent manner. Data show that this drug is more effective after 48 h of treatment compared to 72 h, although it cannot be excluded that over longer periods of time, cordycepin is degraded by falling below the efficacy threshold at the lowest treatment concentrations. In addition, it seems that after 2 days of treatment with cordycepin, an additional dosage of the drug is required to recover the efficacy (Figure 2).

### 3.3. Expression Analysis Revealed WIF1 Re-Expression and MYC Down-Modulation in MUTZ-2 Cells after Treatment with Cordycepin

To address relevant expression patterns in the Wnt pathway transcriptome, we performed targeted AmpliSeq analysis in cordycepin-treated MUTZ-2 cells. Four groups of cells were analyzed in triplicates (Repl. 1–3); the first group is the untreated cells, and groups 2, 3 and 4 were treated with increasing concentrations of 50, 100 and 200 μM cordycepin, respectively, and analyzed after 6 and 24 h of treatment.

**Figure 3:**
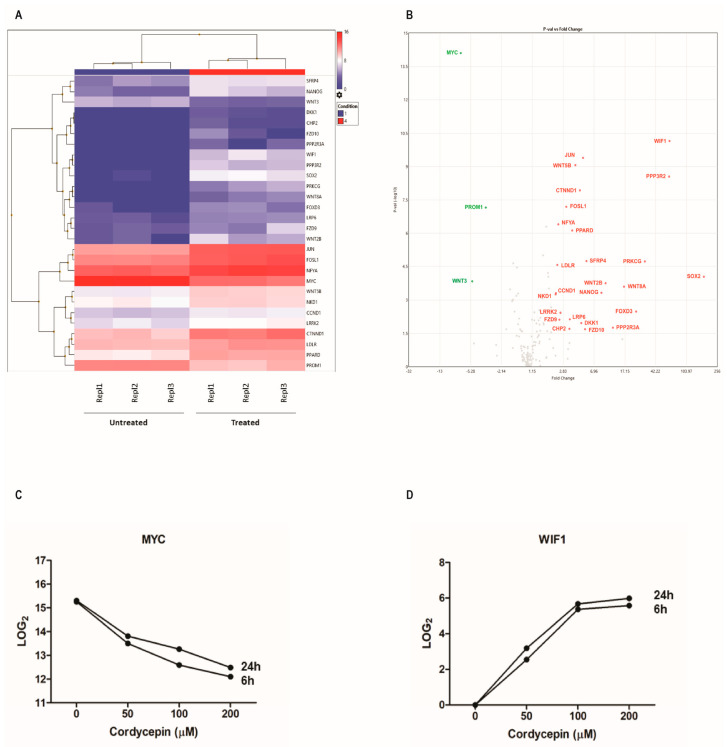
WNT−targeted RNA sequencing for gene expression analysis by AmpliSeq NGS comparing untreated (control) cells and MUTZ−2 cells treated with cordycepin. (**A**) Representative correlation heatmap between untreated (Repl. 1–3) samples and samples treated with 200 μM cordycepin for 24 h (Repl. 1–3). The heatmap shows the differentially expressed genes in the two groups considering only those with an adjusted *p*-value < 0.05 and a |log2FC| ≥ 2. Samples (columns) and genes (rows) are clustered using hierarchical clustering. The heatmap shows the 28 SDE genes for each considered sample, and intensity indicates the ratio of mRNA expression levels detected. *WIF1*, *PPP3R2*, *PRKCG*, *NANOG*, *LDLR* and *SOX2* expression is upregulated in the treated samples, whereas *MYC* and *PROM1* are strongly downregulated in the same group compared to the controls. (**B**) Volcano plot for the control and treated MUTZ-2 cells (*p*-value vs. fold-change ratio) shows the SDE genes highlighted in red dots (genes with BH-adjusted *p*-value ≤ 0.05 and a log2FC ≥ 2). The horizontal dotted line corresponds to *p*-value = 0.05 and the two vertical dotted lines to log2FC = ±2. The expression levels (average log2) of the two genes, *MYC* (**C**) and *WIF1* (**D**), most differentially expressed, among the 8 identified SDEs. The graphs show the expression values of the two most differentially expressed genes among the 8 identified SDEs genes, *MYC* (**C**) and *WIF1* (**D**) at different concentrations (50, 100, 200 μM) of cordycepin detected at 6 h and 24 h after treatment. Expression values for each point are detailed in Table S2.

Targeted RNA sequencing technology enables a sensitive detection of significantly differentially expressed (SDE) genes of the Wnt pathway including upregulation of *WIF1*, *PPP3R2*, *PRKCG*, *NANOG*, *LDLR*, *DKK1* and *SOX2,* and downregulation of *MYC* and *PROM1* was observed in the treated cells compared to the untreated ones (Figure 3, Table 1).

**Figure 4:**
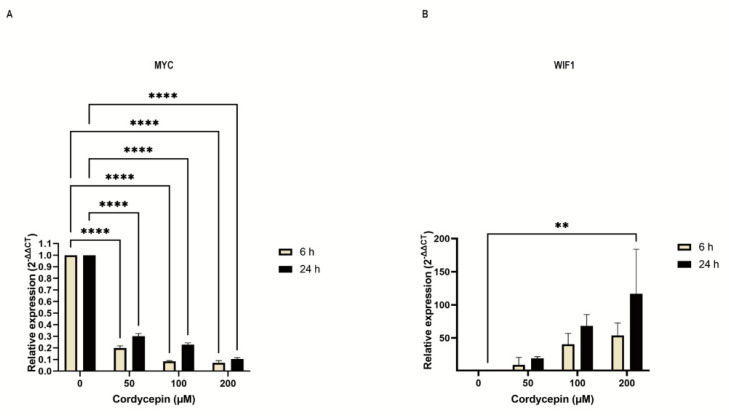
Quantitative expression analysis of MYC and WIF1 genes. MUTZ−2 cells were cultured with increasing concentrations of cordycepin, and RNA was extracted after 6 h and 24 h. Subsequently, RT−qPCR analysis was performed for two indicated WNT signaling effector genes, MYC (**A**) and WIF1 (**B**) in the MUTZ−2 cell line. Undetermined Ct for untreated sample in WIF1 was considered as 40 Ct for the calculations. Bar graphs represent the mean ± SD of at least three independent experiments. Two-way ANOVA with Tukey’s multiple comparisons test was used for statistical analysis; ** *p* < 0.01, **** *p* < 0.0001.

In addition, real-time qPCR was performed to confirm the result obtained by Ampliseq transcriptomic analysis for the two major SDE genes, including WIF1, which is a relevant antagonist of the Wnt signaling and MYC, a major agonist induced by the Wnt pathway (Figure 4A,B). It is notable that WIF1 transcripts are undetectable in MUTZ−2 cells, and cordycepin exposure results in a rapid dose-dependent activation of gene expression. The expression-inducing effect for WIF1 after cordycepin exposure was also detectable by endpoint RT−PCR in MUTZ−2 after 24 h of treatment at increasing cordycepin concentrations (50,100, 200 µM) (Figure S1).

#### Immunoblot Blot Analysis on MYC Protein following Cordycepin Exposure

**Figure 5:**
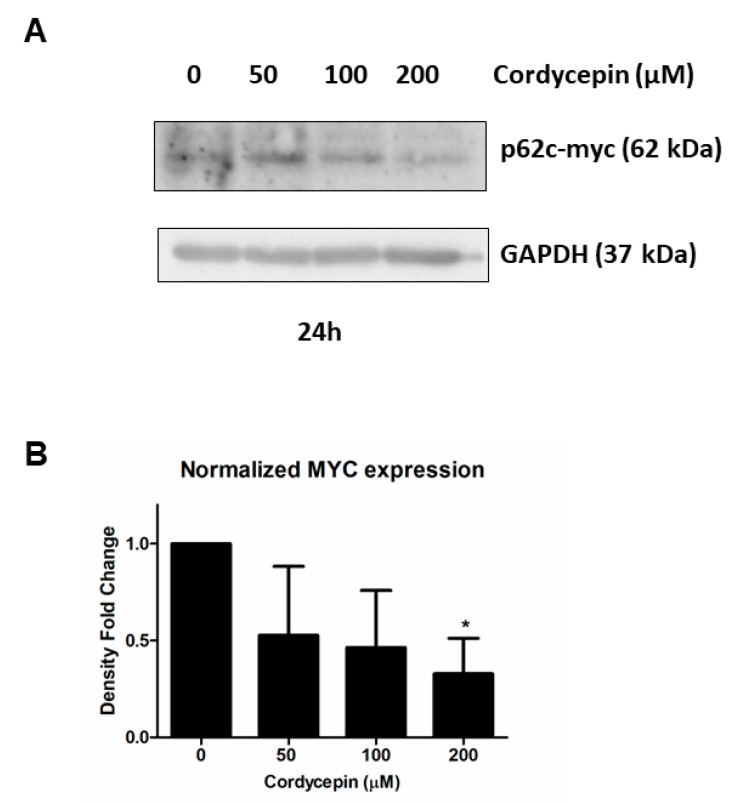
Immunoblot analysis of MYC in MUTZ−2. (**A**) Representative immunoblot on protein extracts of cultured MUTZ−2 cells with increasing concentrations of cordycepin (50, 100 and 200 μM) after 24 h. Immunoblot analysis was performed in three replicates for each drug concentration with antibodies against MYC and GAPDH introduced as equal loading control. (**B**) Bar graph represents the densitometric readings of three MYC immunoblot replicates normalized to GAPDH. Bar graphs represent the mean ± SD of three independent experiments. One-way ANOVA with Dunnett’s test was used for statistical analysis; * *p* < 0.05. In Figure S2: Original western blots.

In view of the relevant observation suggesting the down-modulation of MYC at the transcriptional level following exposure to increasing doses of cordycepin, we evaluated whether this observation was also confirmed at the protein level. For this purpose, we performed three replicas of immunoblot analysis for MYC in MUTZ−2 cell line, treated with increasing concentrations of cordycepin (50, 100, 200 µM) at 24 h to analyze the level of MYC protein (Figure 5A and Figure S2). Consistent with previous results, we observed a decrease in MYC protein (p62c-myc) level in a dose-dependent manner after 24 h of exposure to increasing doses of cordycepin. Consistent with previous results, we observed a significant decrease in MYC level at the highest dose of cordycepin (200 µM) after 24 h (Figure 5B, Table S3).

## 4. Discussion

There is a strong need to develop strategies that eliminate AML−LSCs through the specific interference of the molecular functions that support them without affecting normal hematopoietic stem cells (HSCs). Several works have highlighted the crucial role of the Wnt/β-catenin pathway in activating an early stage of myeloid regeneration pathway in LSC [2,5,6,7,8,9]. Targeting the LSC is a potential adjuvant treatment for acute myeloid leukemia to overcome resistance to therapy and relapse [29,36]. Previous studies reported that cordycepin induces protein degradation of β-catenin via GSK−3β signaling activation, promotes leukemia apoptosis and eliminates leukemia stem cell activity [31,32,33,37]. However, the full picture by which cordycepin attenuates the Wnt/β−catenin signaling in leukemia has only been partially investigated.

The aim of the present study is to elucidate the molecular circuits through which cordycepin interferes with the Wnt pathway transcriptional program and at the same time highlighting details of the mechanisms that maintain the abnormal autocrine Wnt activity in LSC. Therefore, we evaluated the capacity of cordycepin to reduce cell viability and induce apoptosis, and the Wnt-associated transcriptomic response in the leukemic stem cell model MUTZ−2 cell line, which is enriched for AC133^+^ marker [35], and in primary AC133^+^ and MNC AML cells. The results demonstrated that cordycepin exerted a marked inhibitory effect on the MUTZ−2 and AC133^+^ cell proliferation, strongly inducing apoptosis in a dose-dependent fashion.

As previously reported, expression of DKK1, a Wnt/β-catenin antagonist, was stimulated in the mesenchymal stem cells after cordycepin treatment, suggesting a possible paracrine inhibition on the Wnt pathway [32]. In the present study, we provide evidence for another hint of how cordycepin can interfere in the Wnt/β-catenin pathways. The transcriptomic analysis revealed the upregulation of the Wnt inhibitor factor 1 (WIF1), a major negative regulator of Wnt signaling, after cordycepin treatment [38]. In different tumors, a hyper-methylation of the CpG island of the promoter of WIF1 is present, leading to WIF1 silencing and the consequently enhanced activation of the Wnt signaling [39,40]. Accordingly, the re-expression of WIF1 could be an important step in marking the regaining control of the Wnt pathway. The molecular mechanisms by which WIF1 regains expression after cordycepin treatment is still unclear, however. Liu et al. [41] investigated how adiponectin (ADN), an adipokine, is able to promote WIF1 expression by stimulating epigenetic activation. It is proposed that after treatment with ADN, specificity protein 1 (Sp1), a transcriptional factor negatively involved in WIF1 regulation, and its target DNA methyltransferase 1 (DNMT1) decrease in protein concentration. Subsequently, the number of WIF1 methylated CpG islands is decreased, while WIF1 transcripts and proteins are upregulated, suggesting a correlation between ADN concentration and *WIF1* expression [41]. Of interest, treatment in mice with extracts of a newly discovered *Cordyceps* species exhibited elevated levels of adiponectin within the plasma [42]. The re-establishment of WIF1 levels leads to the inhibition of the canonical Wnt pathway and the consequent downregulation of its target genes. One of these downstream genes is the proto-oncogene *MYC*. Through its dimerization with MAX, it has a role as a transcriptional factor of numerous other target genes as well as being involved in the regulation of proliferation, apoptosis, differentiation and cell cycle [43,44,45]. In AML, *MYC* is commonly overexpressed due to different mechanisms and represents a poor prognostic factor giving rise to therapy resistance and risk of relapse [25,26]. *MYC* deregulation plays different roles in genomic instability, immortalization and escape from the immune system, and it is also involved in the inhibition of myeloid differentiation [43,46,47]. Its downregulation can impact the cell’s proliferation and fate, leading to apoptosis. Hence, it was suggested to target *MYC*, directly or indirectly, as an AML treatment. Increasing numbers of studies have focused on targeting *MYC*; however, several difficulties were found since *MYC* is a downstream gene of different pathways. Moreover, the small molecules used to interfere with the fundamental interaction between MYC and MAX showed unclear results [43,44]. We show, in this study, that cordycepin has the ability to reduce *MYC* expression and affects the protein level at higher concentrations. MYC protein degradation is modulated by the activity of ERK1 and GSK3β [48], both of which are impacted by cordycepin; it is therefore likely that following exposure to cordycepin, the level and stability of the MYC protein, tightly regulated by the ubiquitin–proteasome system (UBS) [49], is affected in a time- and concentration-dependent manner, and/or that may be determined by a potential effect on the translational control, as suggested in a previous study [50].

**Figure 6:**
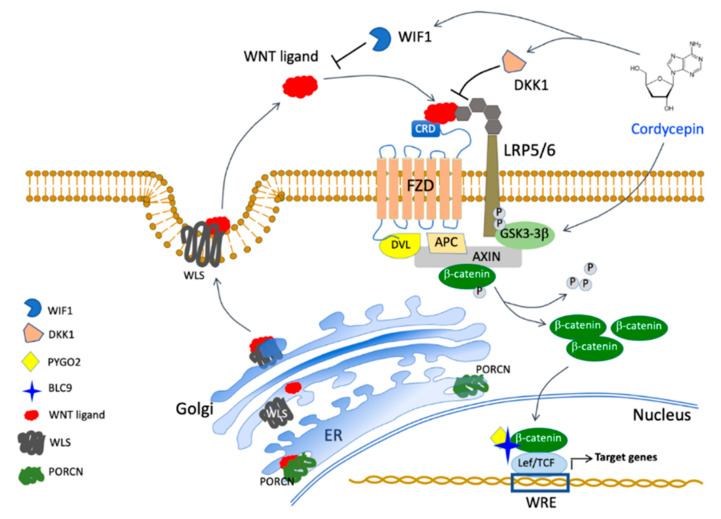
Speculative model of the cordycepin action on Wnt signaling. Cordycepin selectively reduces β-catenin stability via GSK-3β [50] and induces re-expression of the major negative regulators WIF1 and DKK1 inhibiting the expression of MYC in leukemia cells. WRE: Wnt-responsive elements; WLS: Wnt-Wntless binding.

Of interest, we observed the increase in *SOX2* expression following exposure to increasing doses of cordycepin. The significance of this upregulation will require specific evaluation, but of interest is the observation that the overall survival was significantly better for AML patients with high SOX2 levels [51]. In conclusion, cordycepin seems to affect LSCs at different levels interfering with Wnt/β−catenin, necessary for self-renewal maintenance, through the re-activation of *WIF1* and *DKK1* besides the subsequent suppression of *MYC* expression (Figure 6).

Further investigation is needed to identify the molecular mechanisms driving *WIF1* re-expression. Furthermore, the evaluation of the in vivo effect of cordycepin is necessary since it is rapidly metabolized by adenosine deaminase (ADA) [32] and by the acid conditions of the stomach [52].

## 5. Conclusions

This study confirmed that cordycepin has an effective ability to suppress proliferation, inducing apoptosis of leukemia cells through a pleiotropic mechanism involving different molecular components of Wnt signaling [31,32,33,53]. Thus, the efficacy of cordycepin, highlighted in this and previous studies [31,32,33,54] suggests the possibility of its combined use in an integrative medicine context for the treatment of AML as an effective resource for LSC eradication, also as a result of the unsatisfactory impact obtained by other Wnt inhibitors [30]. Therefore, improving the pharmacological formulation of cordycepin or providing its injection with ADA inhibitor will permit a longer effect and the drug’s ability to reach the affected district. Nonetheless, cordycepin has long been used as a food supplement in traditional Chinese medicine, suggesting low side effects and a good safety profile. This study supports the potential use of cordycepin in integrative oncology [55] as an adjuvant for AML treatment due to its capacity to target leukemia stem cells as well as its ability to act specifically on them, reducing cell viability and stimulating apoptosis of LSCs.

## Figures and Tables

**Table 1:** Significantly differentially expressed Wnt-Genes by Ampliseq transcriptomic analysis following cordycepin treatment. The table shows the correlation between untreated samples (n = 3) and samples treated with 200 μM cordycepin after 24 h (n = 3) from the AmpliSeq transcriptomic analysis. The selected genes shown in the table are SDE genes in the two groups considering only those with an adjusted *p*-value < 0.05 and a |log2FC| ≥ 2.

Gene Expression Avg (log2)	Untreated	200 µM COR	Fold Change
**MYC**	15.3	12.5	−7.1
**PROM1**	11.7	9.9	−3.4
**WIF1**	0.0	6.0	63.3
**PPP3R2**	0.0	6.0	62.7
**PRKCG**	0.0	4.5	30.7
**DKK1**	0.0	2.3	4.8
**NANOG**	3.3	6.4	8.6
**SOX2**	0.0	7.4	172.2

## Data Availability

The data presented in this study are available in this article and Supplementary Material.

## Supplementary Materials

The following supporting information can be downloaded at: https://www.mdpi.com/article/10.3390/cancers15153931/s1.
