# Supplementary material for: Cordycepin (3′dA) Induces Cell Death of AC133+ Leukemia Cells via Re-Expression of WIF1 and Down-Modulation of MYC
Source: Cancers (Basel). 2023 Aug 2;15(15):3931. doi: 10.3390/cancers15153931 (PMC10417454; doi:10.3390/cancers15153931)
Supplement: Supplementary file 1 [file cancers-15-03931-s001.zip › cancers-2421691-supplementary.pdf]

Primer	Sequence 5'→3'
GAPDH Forward	ACA ACA GCC TCA AGA TCA TCA G
GAPDH Reverse	GGT CCA CCA CTG ACA CGT TG
MYC Forward	CCC GCT TCT CTG AAA GGC T
MYC Reverse	CTC CTC CTC GTC GCA GTA G
WIF1 Forward	CAT CTG CCC ACC TGG ATT CT
WIF1 Reverse	CCA GGG TAG AAA CAG GTC CC

Table S1. List of primers used in quantitative real-time PCR (qRT-PCR) analysis.

Gene expression Avg (Log2)	Cordycepin(µM)							
	Untreated		50		100		200	
	6h	24h	6h	24h	6h	24h	6h	24h
**MYC**	15.3	15.3	13.5	13.8	12.6	13.3	12.1	12.5
**WIF1**	0.0	0.0	2.6	3.2	5.4	5.7	5.6	6.0

Table S2. Expression levels of MYC and WIF1 obtained by AmpliSeq WNT-targeted RNA next generation sequencing. Expression levels (average Log2) of MYC and WIF1, the most relevant genes among the 8 highlighted SDE, analyzed for different concentrations (50, 100 and 200 µM) of cordycepin at 6h and 24h after the treatment.

REPLICATE	COR (μM)	Adj. Volume MYC	Volume MYC	Lane % MYC	Adj. Volume GAPDH	Volume GAPDH	Lane % GAPDH	MYC NORMALIZED DENSITY	DENSITY FOLD CHANGE
1	not treated	897396	2029324	97,751935	325035	921465	97,410654	2,76	**1**
1	50 uM	861135	2014947	98,119947	337716	864252	97,464935	2,55	**0,92**
1	100 uM	872228	1867320	98,389185	393320	944000	97,202452	2,22	**0,80**
1	200 uM	617292	1974024	97,531426	424488	1064440	97,341772	1,45	**0,53**
2	not treated	1451775	6003309	92,079551	178698	904254	96,625896	8,12	**1**
2	50 uM	1022628	3208314	90,479717	300840	945990	97,626511	3,40	**0,42**
2	100 uM	715407	2589180	90,767878	314424	997656	96,908055	2,28	**0,28**
2	200 uM	550562	2420562	84,496973	407115	987390	97,112495	1,35	**0,17**
3	not treated	2976246	3873282	99,804231	634016	1150720	99,204851	4,69	**1**
3	50 uM	738854	2334889	96,709272	666080	1140608	98,902617	1,11	**0,24**
3	100 uM	957759	2647623	96,608082	661395	1201095	99,331877	1,45	**0,31**
3	200 uM	859740	2664060	95,124501	623968	1131826	99,255186	1,38	**0,29**

Table S3.

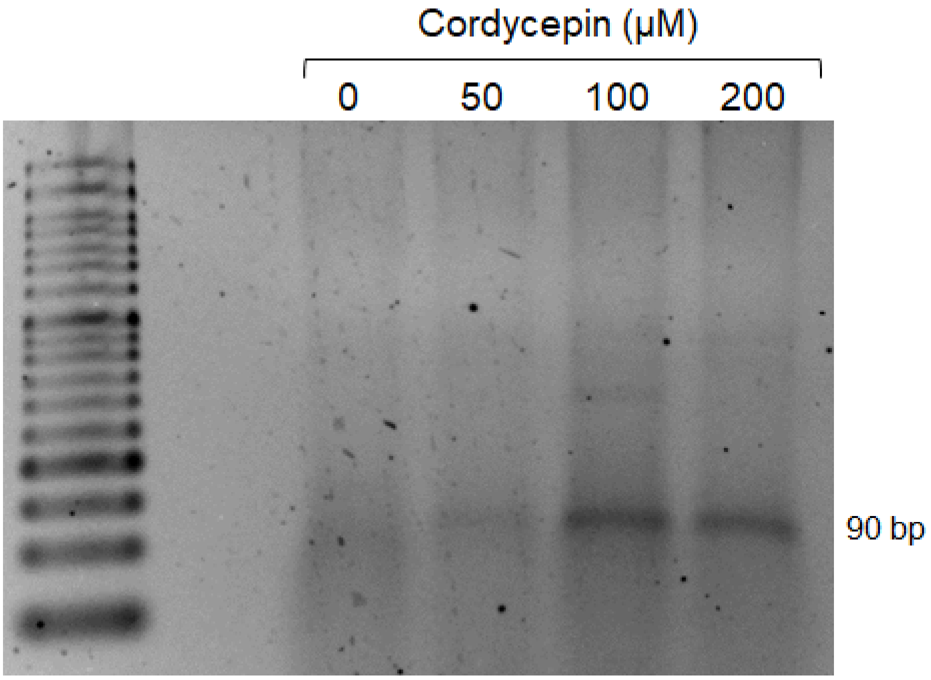

Figure S1. RT-PCR endpoint for WIF1 in MUTZ-2.

RT-PCR endpoint performed in MUTZ-2 after 24h treatment with different concentrations of cordycepin. WIF1 expression increases after treatment with 50 µM of the drug. First lane shows 50 bp DNA ladder.

REPLICATE 1

p62c-myc (62 kDa)

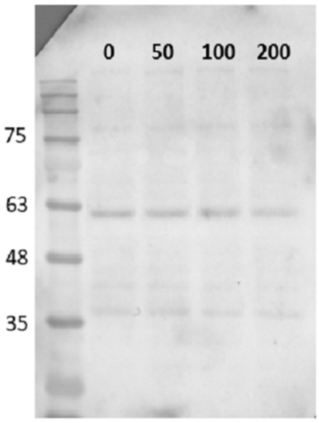

GAPDH (37 kDa)

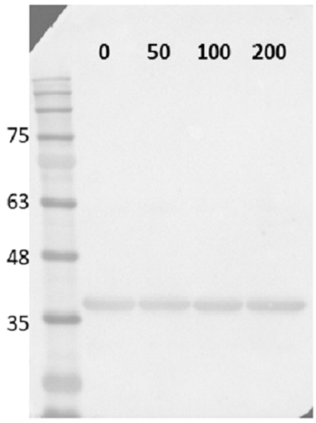

REPLICATE 2

p62c-myc (62 kDa)

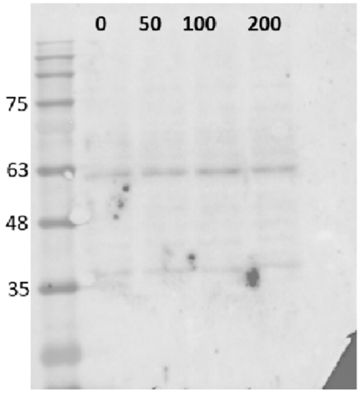

GAPDH (37 kDa)

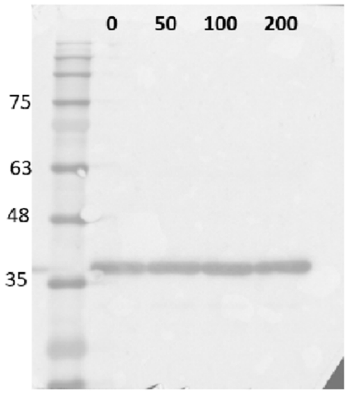

REPLICATE 3

p62c-myc (62 kDa)

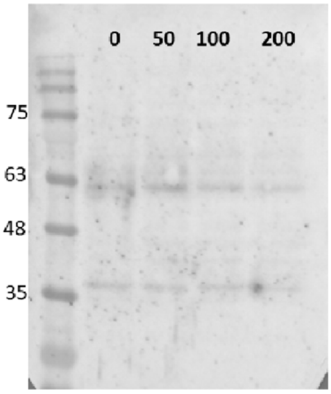

GAPDH (37 kDa)

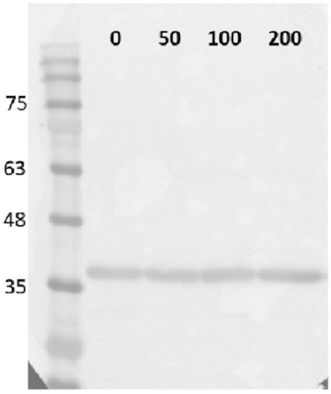

Figure S2. Immunoblot analysis of MYC in MUTZ-2. (A-C) MUTZ-2 cells were cultured with increasing concentrations of cordycepin (50, 100 and 200 μM), and then protein was extracted from the cells with RIPA buffer after 72 h. Immunoprecipitation was done for MYC by using magnetic beads and then Immunoblot analysis has been performed with antibodies against MYC (1:1000) and using GAPDH (1:1000) as equal loading control. Protein marker: Opti-Protein XL Marker/Ladder 10-245 kDa. **(B-D)** Spot separation made by the software UVITEC-1D connected to the instrument UVITEC Alliance HD 6, Cambridge for the calculation of the density of each band obtained after Immunoblot analysis of MYC in MUTZ-2. Density values obtained for MYC have been then normalized with the loading control protein GAPDH. The threshold value used in the graph is 0.

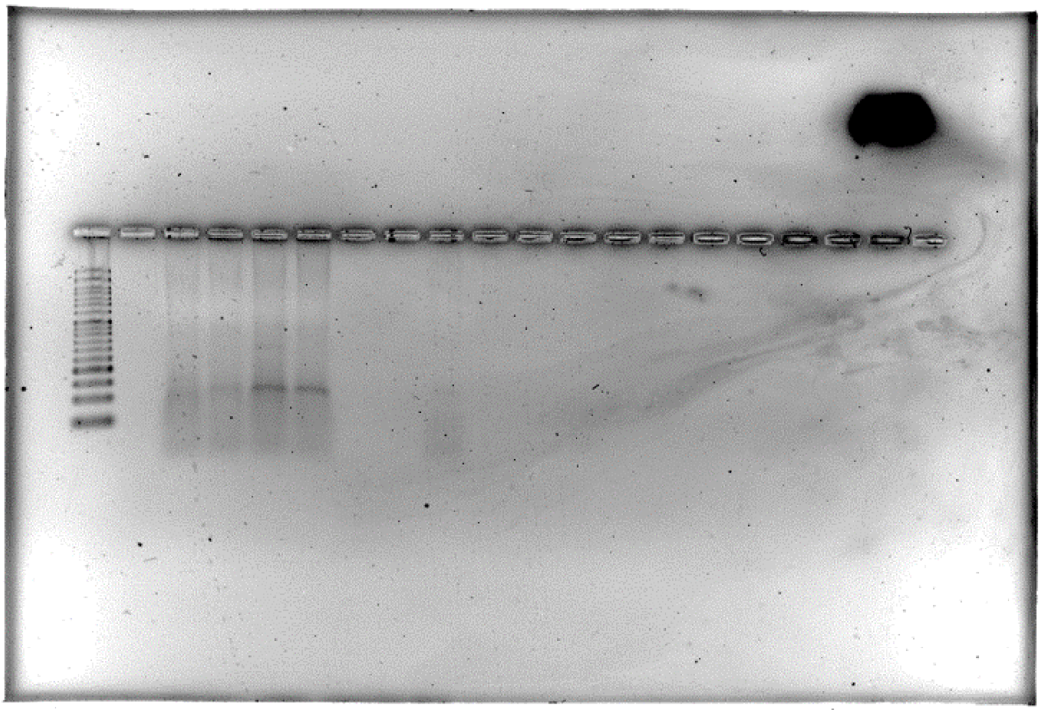

Figure S1 uncropped
